# Rapid cell division of *Staphylococcus aureus* during colonization of the human nose

**DOI:** 10.1186/s12864-019-5604-6

**Published:** 2019-03-20

**Authors:** Anna K. Szafrańska, Vera Junker, Matthias Steglich, Ulrich Nübel

**Affiliations:** 1 Leibniz Institute DSMZ - German Collection of Microorganisms and Cell Cultures, Inhoffenstr. 7B, 38124 Braunschweig, Germany; 2 German Center for Infection Research (DZIF), Braunschweig site, Germany; 3 Braunschweig Integrated Centre of Systems Biology (BRICS), Technical University Braunschweig, Braunschweig, Germany

**Keywords:** Commensal bacteria, Nasal microbiome, Replication rate, Generation time, In-vivo growth dynamics, Metagenome sequencing, Mutation rate, Mutation accumulation

## Abstract

**Background:**

*Staphylococcus aureus* is an important opportunistic pathogen and a commensal bacterium, thriving in the nasal cavities of 20% of the human population. Little is known about the dynamics of asymptomatic colonization and the occasional transition to infectious disease.

**Results:**

In this study, we inferred that *S. aureus* cells replicate every one to three hours on average while colonizing the human nose, based on two independent lines of genomic evidence. First, we collected nasal swab samples from human subjects, extracted and sequenced metagenomic DNA, and analyzed the distribution of sequencing coverage along the staphylococcal chromosome. Calibration of this data by comparison to a laboratory culture enabled measuring *S. aureus* cell division rates in nasal samples. Second, we applied mutation accumulation experiments paired with genome sequencing to measure spontaneous mutation rates at a genome scale. Relating these mutation rates to annual evolutionary rates confirmed that nasal *S. aureus* continuously pass several thousand cell divisions per year when averaged over large, globally distributed populations and over many years, corresponding to generation times of less than two hours.

**Conclusions:**

The cell division rates we determined were higher than the fastest documented rates during fulminant disease progression (in a mouse model of systemic infection) and much higher than those previously measured in expectorated sputum from cystic fibrosis patients. This paper supplies absolute in-vivo generation times for an important bacterial commensal, indicating that colonization of the human upper respiratory tract is characterized by a highly dynamic equilibrium between bacterial growth and removal.

**Electronic supplementary material:**

The online version of this article (10.1186/s12864-019-5604-6) contains supplementary material, which is available to authorized users.

## Background

Opportunistically pathogenic bacteria colonize their hosts for extended periods, causing disease symptoms only occasionally. The processes that cause progression to disease from colonization are of great interest, since an improved assessment of an individual’s risk of infection would enable early intervention and the development of targeted therapies. Due to the complexity of host-pathogen interactions, mathematical modeling will likely be needed to explore and understand their dynamics [1]. For adequate parameterization, however, modeling will require quantitative data on factors that govern these interactions, and among these, microbial population density and the in vivo growth rate commonly are considered central [1–3].

Very little data is available on the growth rates of pathogenic bacteria in the colonized or infected host. Previously reported in-vivo growth rates had been based on monitoring frequency changes of genetically tagged pathogens, following their inoculation into animal models [4–7]. For example, when mice were infected with a *Streptococcus pneumoniae* strain which carried a temperature-sensitive, antibiotic-resistance plasmid, the observed dilution of that plasmid during bacterial growth provided evidence for generation times of 2–3 h [4]. For clinical relevance, however, in vivo data collected from human subjects is to be preferred over that from animal models [3]. Recently, the pattern of DNA sequencing coverage along bacterial genomes was proposed to provide a quantitative measure of microbial growth [8, 9]. Replication of the circular bacterial chromosome proceeds bi-directionally from the origin towards the terminus of replication. During DNA replication, therefore, the DNA copy number is higher at the origin of replication than at the opposite site, and this will be reflected in the coverage of genomic sequencing. Intriguingly, the peak-to-trough ratio (PTR) of DNA sequencing coverage for a bacterial population can be retrieved by high-throughput sequence analysis of complex, metagenomic DNA, which had been sampled from, for example, human mucosa, skin, or stool [8–10].

*Staphylococcus aureus* is a classic opportunistic pathogen. This bacterium may cause various infections, ranging from self-limiting skin infections to life-threatening pneumonia, bacteremia and endocarditis [11]. In particular, methicillin-resistant *S. aureus* (MRSA) is a notorious cause of healthcare-associated and community-associated disease [11]. Primarily, however, *S. aureus* is a colonizer of the nasal vestibule, and infection commonly is preceded by colonization [12]. Even though symptom-free carriage only rarely proceeds to serious infection, the overall burden of disease is high due to a very high carriage rate. Globally, 20% of the human population is colonized with *S. aureus* [13].

Host-pathogen interactions during symptom-free nasal carriage and the physiological conditions which *S. aureus* experiences during colonization of the nasal cavity are incompletely understood. *Staphylococcus aureus* may adhere to the epithelium of the anterior nostrils and to cells of the inner nasal cavity. Adherence gets mediated by several proteins (including clumping factor B) and glycopolymers (teichoic acids) on the surface of *S. aureus* cells, which bind to specific ligands of the nasal epithelium [14]. In rodent models, adhesin-deficient *S. aureus* mutants got eliminated rapidly, suggesting that adherence was important for maintained colonization [15, 16]. To reduce contact with microorganisms, the epithelia of the nasal cavity and the nasopharynx are covered with a layer of viscous mucus. The mucus traps airborne particles, including microorganisms, and through ciliary action continuously flows towards the oropharynx, to clear any trapped material [17]. In addition to forming a physical barrier to microbial colonization, the mucus provides a matrix for antimicrobial molecules, including immunoglobulin A, neutrophil-derived peptides, and epithelial defense peptides [18, 19]. Nutrients for bacterial growth are less abundant in the respiratory tract than in the gastrointestinal tract, since their supply solely depends on secretions from the host [20]. The host’s airway epithelia even actively deplete glucose from the airway surface fluid to suppress microbial proliferation [21]. Cultured strains of *S. aureus*, however, were able to grow on dilute low-molecular weight compounds found in human nasal secretions [20]. In addition, *S. aureus* can utilize for growth some of the oligosaccharides that cover mucin glycoproteins, which are the major macromolecular constituent of mucus and which also decorate the surface of epithelial cells [22]. Other constituents of the resident microbiome may cooperate in the utilization of complex glycoproteins [23] or compete for nutrients and receptor space [24].

*Staphylococcus aureus* is among the fastest evolving bacteria. Population genomic analyses, on the basis of temporally structured samples of *S. aureus* genome sequences, indicated that the staphylococcal chromosome continuously accumulates 2 × 10^− 6^ base substitutions per nucleotide per year (Table 1 and references therein). Remarkably, this evolutionary rate was determined as an almost uniform, species-specific trait across multiple studies, despite differences in sampling schemes and analysis methods (Table 1 and references therein). For as yet unknown reasons, this rate is about ten times higher than the evolutionary rates found in *Mycobacterium tuberculosis* [25], *Clostridioides difficile* [26], *Klebsiella pneumoniae* [27], or *Escherichia coli* [28]. Generally, organisms with shorter generation times evolve faster, as they replicate their genomes more frequently and therefore accumulate more mutations per unit time. A generation-time dependency of the molecular evolutionary rate has been observed repeatedly for animals (e. g., rodents vs. primates [29]) and plants (annual vs. perennial plants [30]). Similarly, a lower long-term evolutionary rate was recently reported for bacteria that are able to form dormant, non-reproductive states (endospores) in comparison to related species that do not have this ability [31]. Relating the short-term evolutionary rate (at which mutations accumulate per year) to the spontaneous mutation rate (at which mutations occur per generation) should yield an estimate of the number of generations passing per year [29, 32], provided that the majority of mutations observed in natural populations at the basis of evolutionary rate estimates were selectively neutral. Even though evolutionary rate estimates for bacteria commonly have not been restricted to synonymous mutations, such neutrality appears a good approximation for *S. aureus* population genomics, since the vast majority of newly introduced base substitutions remain detectable in *S. aureus* populations for decades [33–35]. These substitutions may get removed by purifying selection over much longer time scales eventually [36], suggesting that they are in fact weakly deleterious over the long term, or nearly neutral over the short term. Purging of weakly deleterious mutations may lead to a slight underestimation of the short-term evolutionary rate [37], and of the cell division rate accordingly.

**Table 1:** Previously reported short-term evolutionary rates from *S. aureus* clonal lineages

Clonal lineage ^a^	Evolutionary rate (95% confidence intervals) [Base substitutions per nucleotide per year]	Reference
CC22	1.30 × 10^− 6^ (1.26 × 10^− 6^ - 1.40 × 10^− 6^)	[35]
CC30	1.42 × 10^− 6^ (1.04 × 10^− 6^ - 1.80 × 10^− 6^)	[80]
ST225 (CC5)	2.00 × 10^− 6^ (1.20 × 10^− 6^ - 2.90 × 10^− 6^)	[34]
CC398	1.68 × 10^− 6^ (0.70 × 10^− 6^ - 2.49 × 10^− 6^)	[38]
CC8	1.22 × 10^− 6^ (0.60 × 10^− 6^ - 1.86 × 10^− 6^)	[81]
ST239	1.60 × 10^− 6^ (1.20 × 10^− 6^ - 2.00 × 10^− 6^)	[82]
ST239	3.30 × 10^− 6^ (2.50 × 10^− 6^ - 4.00 × 10^− 6^)	[33]

^a^ *CC* clonal complex, *ST* sequence type

In this study, we estimated the in vivo cell division rate of *S. aureus* during colonization of the human nose on the basis of two independent lines of genomic evidence. First, we used mutation accumulation experiments paired with genome sequencing to measure mutation rates at a genome scale. By relating these mutation rates to annual evolutionary rates, which had previously been determined on the basis of temporally structured samples of staphylococcal genome sequences [33, 35, 38], we estimated average *S. aureus* cell division rates over large, globally distributed populations and over many years. Second, we collected nasal swab samples from human subjects and sequenced extracted metagenomic DNA. The distribution of sequencing coverage along the staphylococcal chromosome provided a measure of *S. aureus* cell division rates in sampled individuals.

## Results

### Mutation rates from fluctuation analyses

**Fig. 1:**
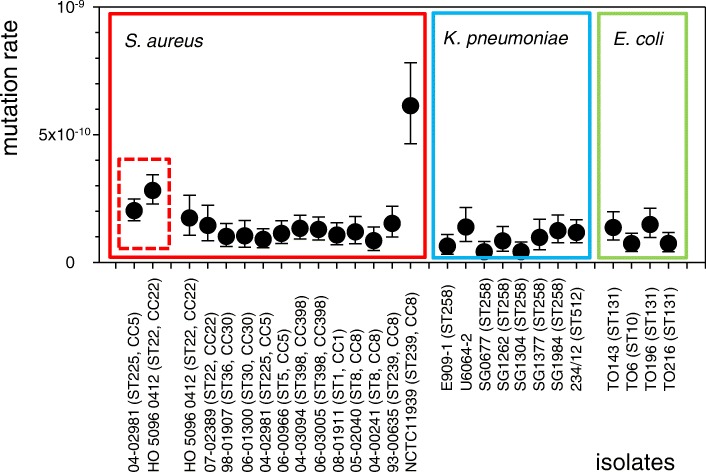
Spontaneous mutation rates for bacterial isolates from three species. Mutation rates from mutation accumulation experiments and their 95% confidence intervals are indicated on the very left (dashed frame). For mutation rates from fluctuation analyses, means from duplicate measurements and 95% confidence intervals are shown

We performed fluctuation analyses for 13 isolates of *S. aureus* (Additional file 1: Table S1). Isolates had been chosen to maximize phylogenetic diversity, and we included both, methicillin-resistant *S. aureus* (MRSA) and methicillin-susceptible isolates from each of six clonal complexes that presently dominate global MRSA populations (Additional file 1: Table S1, Fig. 1). For each of these particular clonal lineages, population genomic analyses had been performed previously and, accordingly, short-term evolutionary rates are available from the literature (Table 1, see references in Table).

By sequencing *rpoB* gene segments from 100 rifampicin-resistant mutants, we found that 17 unique point mutations had caused rifampicin resistance in *S. aureus* (Additional file 2: Table S2), which is consistent with previous reports [39, 40]. Accordingly, by dividing the phenotypic rate by the target size, we estimated that mutation rates were 0.8 × 10^− 10^ (95% confidence interval, 0.5 × 10^− 10^ to 1.4 × 10^− 10^) to 1.7 × 10^− 10^ (95% confidence interval, 1.1 × 10^− 10^ to 2.6 × 10^− 10^) mutations per nucleotide per generation in most isolates (median, 1.2 × 10^− 10^), and up to 6.1 × 10^− 10^ (95% confidence interval, 4.6 × 10^− 10^ to 7.8 × 10^− 10^) mutations per nucleotide per generation in NCTC11939 (ST239; Fig. 1). Hence, mutation rates were very uniform among different *S. aureus* isolates, with the exception of a single isolate (NCTC11939) affiliated to sequence type ST239, which displayed an approximately five-fold elevated rate (Fig. 1); of note, the mutation rate of another ST239 isolate (93–00635) was inconspicuous (Fig. 1). Mutation rates were not noticeably different between MRSA and methicillin-susceptible isolates (*P* > 0.05; Fig. 1).

For comparison, we determined mutation rates for eight isolates of *K. pneumoniae* and four isolates of *E. coli* (Additional file 1: Table S1). These isolates represented sequence types for which evolutionary rates had been determined previously, including multiresistant, pandemic strains *K. pneumoniae* ST258 and *E. coli* ST131 (Additional file 1: Table S1). Median mutation rates of *K. pneumoniae* and *E. coli* were not significantly different from those of *S. aureus* (*P* > 0.05, Fig. 1).

### Mutation rates from mutation accumulation experiments

We performed mutation accumulation experiments with two unrelated isolates of methicillin-resistant *S. aureus*, 04–02981 (ST225) and HO 5096 0412 (ST22). These isolates represent two strains which currently predominate MRSA populations in Europe and beyond [34, 35]. From each strain, single colonies of 59 or 62 lines, respectively, were transferred onto fresh agar plates every 24 h to pass 1972 and 2695 cell divisions total (Table 2). Sequencing the genomes from final colonies of each line revealed a total of 97 and 91 mutations, respectively (Table 2, Additional file 3: Table S5 and Additional file 4: Table S6). Similar to previous studies on *E. coli* and *Vibrio shilonii* [41, 42], base substitutions were about 10-fold more common than insertions/deletions, and transitions were more abundant than transversions (Table 2). The ratio of non-synonymous to synonymous base substitutions did not significantly differ from random expectations [41], and mutations were randomly distributed along the *S. aureus* genome (Additional file 3: Table S5 and Additional file 4: Table S6). From these numbers, we calculated similar mutation rates for the two strains of 2.8 × 10^− 10^ mutations per nucleotide per generation (95% confidence intervals, 2.3 × 10^− 10^ to 3.4 × 10^− 10^) and 2.0 × 10^− 10^ mutations per nucleotide per generation (95% confidence intervals, 1.6 × 10^− 10^ to 2.5 × 10^− 10^), respectively (Table 2).

**Table 2:** Results of mutation accumulation experiments for two *S. aureus* strains

Strain (sequence type)	Lines	Transfers	Generations	Ts	Tv	Ins^a^	Del^a^	Mutation rate per nucleotide, μ_MA_	95% confidence interval
04–02981 (ST225)	62	58	1972	71	26	7	5	2.81 × 10^−10^	2.28 × 10^− 10^ - 3.43 × 10^− 10^
HO 5096 0412 (ST22)	59	77	2695	59	32	4	5	2.02 × 10^− 10^	1.63 × 10^− 10^ - 2.48 × 10^− 10^

^a^ Insertion and deletions ≤4 basepairs

### Rate of in vivo cell replication inferred from the rate of evolution

In the genomes from natural *S. aureus* populations, base substitutions accumulate over calendar time (i. e., over weeks to decades) at the short-term evolutionary rate *k*. Dividing *k* by the spontaneous mutation rate *μ*, at which mutations enter the *S. aureus* population per each round of cell division, provides a crude estimate of the number of cell divisions that have elapsed per unit of time, 1/*g* (where *g* is the generation time).

Based on evolutionary rates published previously (Table 1) and mutation rates determined in this study on the basis of mutation accumulation experiments (Table 2), generation times were estimated at 81 min (95% confidence intervals, 60 to 103 min) for *S. aureus* ST22 and 73 min (95% confidence intervals, 41 to 105 min) for ST225. A recently published mutation rate for *S. aureus* ATCC 25923 was higher than our measurements (4.4 × 10^− 10^ mutations per nucleotide per generation; 95% confidence intervals, 3.9 × 10^− 10^ to 4.9 × 10^− 10^) [43], and consequently yielded a longer generation time estimate of 115 min (95% confidence intervals, 70 to 214 min). ATCC 25923 is a control strain used for antibiotic susceptibility tests and several other assays (http://www.lgcstandards-atcc.org/); its MLST sequence type has as yet not been determined.

### Rate of in vivo cell replication inferred from DNA sequencing coverage

**Fig. 2:**
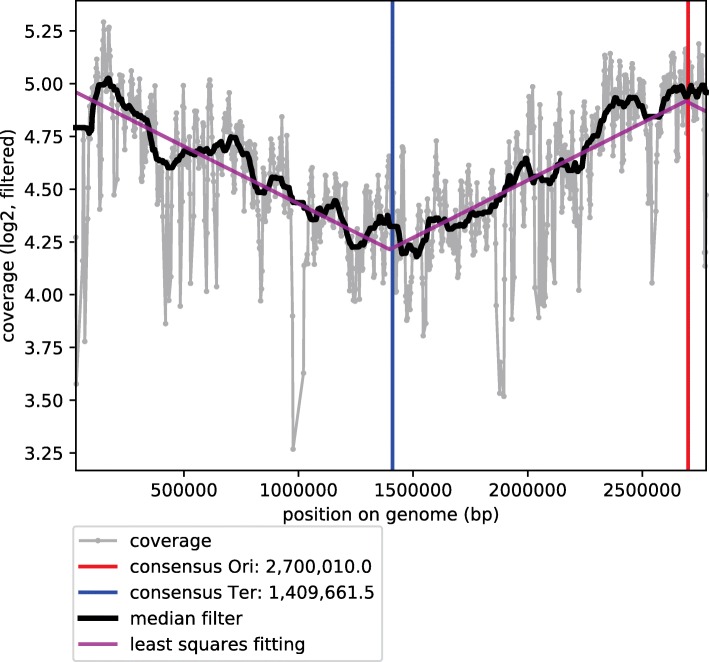
Example to illustrate the pattern of sequencing coverage used to calculate *S. aureus* generation times in vivo. Coverage of sequencing reads from nasal sample no. 32 along the *S. aureus* genome. Genomic locations of the origin (Ori) and terminus (Ter) of replication are indicated. The average sequencing coverage was 23-fold and the PTR was 1.54 (Table 3)

We collected samples from the nasal microbiomes of 46 adult individuals by self-swabbing of their anterior nares, without prior knowledge of the individuals’ *S. aureus* colonization status. By using quantitative PCR, we detected *S. aureus* DNA in ten (22%) of the swabbing samples, with threshold cycles (*C*_*t*_ values) below 30 (*C*_*t*_, 22–29; Table 3), corresponding to 0.3 to 20 pg/μl of *S. aureus* DNA in DNA extracts (not shown). The observed proportion of positively tested samples was consistent with previously reported colonization rates [13]. In contrast, in the majority (67%) of samples, *C*_*t*_ values were ≥ 38, corresponding to < 0.1 fg/μl of *S. aureus* DNA (Additional file 5: Table S3). DNA samples that had been tested positive for *S. aureus* by qPCR were sequenced by applying Illumina technology to achieve ≥14-fold average coverage of the *S. aureus* genome (Table 3). Five-fold sequencing coverage was previously shown to be sufficient for precise calculation of the peak-to-trough coverage ratio (PTR) [9]. In addition, we had included in the sequencing attempt four samples with intermediate *C*_*t*_ values (*C*_*t*_, 31–33), but these failed to produce sufficient numbers of reads from *S. aureus* DNA and achieved < 3-fold coverage of the *S. aureus* chromosome (Table 3). In sequencing datasets with sufficient coverage, 0.3 to 13% of the total sequencing reads could be mapped to an *S. aureus* reference genome (Table 3). In these datasets, sequencing read coverage along the *S. aureus* genome displayed a peak at the origin of genome replication and a trough at the opposite genomic position (Fig. 2). The PTR was between 1.24 and 1.67 for individual samples (median, 1.54; Table 3), indicating that the majority of *S. aureus* cells from the anterior nares had been in a state of replication. Of note, testing several different, unrelated *S. aureus* reference genomes for read mapping showed that the choice of the reference genome had negligible effects on inferred PTR values (deviation, < 3%; data not shown).

**Table 3:** Results from metagenomic sequencing of DNA from nasal swabs

Nasal sample	DNA concentration (ng/μl)	Ct (*gyrB*)	Library concentration (ng/μl)	Total reads	Mapped to *S. aureus* (%)	Average *S. aureus* coverage	PTR	Estimated *S. aureus* doubling time (h)
1	0.16	26	1.95	105,786,161	1,136,111 (1.1)	51	1.57	3.1
2	0.12	27	1.12	55,786,059	2,147,091 (3.8)	85	1.56	3.1
13	0.04	29	0.59	49,621,631	1,738,864 (3.5)	60	1.24	6.9
14	0.06	28	1.32	83,741,570	1,440,622 (1.7)	53	1.55	3.2
18	0.25	28	9.11	154,201,831	681,402 (0.4)	27	1.67	2.2
32	0.37	27	4.78	95,098,143	491,336 (0.5)	23	1.54	3.3
34	0.28	29	8.22	124,493,711	313,424 (0.3)	14	1.46	4.1
44	0.13	23	2.78	10,457,294	958,743 (9.2)	47	1.53	3.4
45	0.29	23	2.70	29,212,511	3,832,248 (13.1)	169	1.53	3.4
46	0.78	22	2.68	22,008,815	1,111,948 (5.1)	54	1.33	5.7
						Median PTR:	1.54	3.4
6	0.09	32	0.36	7,757,779	39,355 (0.5)	1	*n. a.*	*n. a.*
10	*b. d.*	32	0.21	1,979,214	32,857 (1.7)	1	*n. a.*	*n. a.*
19	0.06	31	1.20	6,887,014	65,255 (0.7)	2	*n. a.*	*n. a.*
20	0.06	33	1.21	14,195,854	75,380 (0.5)	3	*n. a.*	*n. a.*

*b. d.* below detection limit, *n. a.* not applicable

**Fig. 3:**
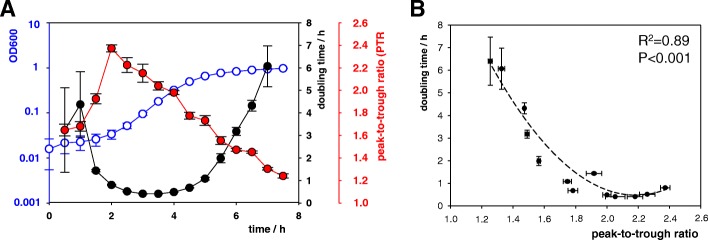
Growth experiment with an *S. aureus* laboratory culture (nasal isolate). Means and standard deviations from triplicate experiments are shown. **a** Growth curve (blue), doubling times (black), and associated PTR from genomic sequencing coverage (red). **b** Interdependency of the PTR from genomic sequencing coverage and doubling time. The dashed line indicates a quadratic polynomial regression fit, and the R^2^ and *P* value for the regression equation are shown

To calibrate growth-rate estimates from PTR, we incubated an *S. aureus* strain as a batch culture and collected a series of samples for DNA sequencing during different phases of bacterial growth. This strain had been isolated from one of the nasal swab samples. Based on optical density measurements during this in-vitro growth experiment, the generation time varied from 24 min. during exponential growth to 364 min. in the early stationary phase (Fig. 3). Sequencing data from each of these culture samples revealed that PTR ranged between 2.4 and 1.2, where a PTR > 2 indicated multifork DNA replication [8]. PTR was strongly correlated with generation time (R^2^ = 0.89, *P* < 0.001; Fig. 3b). Using the regression line from this plot of in-vitro data, we could estimate generation times for *S. aureus* from nasal swabs on the basis of PTR values. Accordingly, we inferred that average *S. aureus* generation times in vivo had been between 2 h and 4 h for the majority of nasal swab samples, and around 6 h to 7 h for two of the samples (median, 3.4 h; Table 3).

## Discussion

### Spontaneous mutation rates

Mutation accumulation experiments combined with genome sequencing are thought to provide the least biased, most realistic, genome-wide mutation rates for bacteria [44, 45]. We found that mutation rates from two unrelated strains of MRSA were 2.0 (95% confidence intervals, 1.6 to 2.5) × 10^− 10^ or 2.8 (95% confidence intervals, 2.3 to 3.4) × 10^− 10^ mutations per nucleotide per generation, respectively. Hence, they were very similar to previously reported mutation rates from wild-type *E. coli* [41] and *Bacillus subtilis* [46], determined by using the same method. In contrast, our fluctuation analyses yielded mutation rates per nucleotide in the gene *rpoB* that were two-fold to three-fold lower. Lower rates from classical fluctuation analyses compared to mutation accumulation are a common observation and have been ascribed to phenotypic delay of antibiotic resistance [41, 47]. Nevertheless, results from our fluctuation analyses indicated that mutation rates were independent from methicillin-resistance and similar among diverse *S. aureus* clonal complexes, with the notable exception of one ST239 isolate (NCTC11939). Of note, the genome sequence from this isolate did not carry any inactivating mutations in genes involved with DNA replication, DNA repair or recombination, which could cause a mutator phenotype [48]. Hence, the reasons for elevated mutation rates in this and in another *S. aureus* isolate investigated recently [43] remain as yet unclear.

Our fluctuation analyses also indicated low variation among mutation rates from multiple isolates of *K. pneumoniae* and *E. coli*, respectively, even though smaller numbers of clonal lineages were investigated for these species. Overall, mutation rates from fluctuation analyses were similar among *S. aureus*, *E. coli*, and *K. pneumoniae*. This finding is in line with mutation accumulation results (for *S. aureus* and *E. coli*) and may suggest similar effective population sizes (i. e., similar strength of genetic drift) for the three species [45]. Importantly, these results indicate that the striking, up to 10-fold differences in annual evolutionary rates between these species are not caused by differential mutation rates.

### Cell division rate of *S. aureus* during nasal colonization

The observation of largely uniform mutation rates from diverse *S. aureus* strains is reminiscent of the uniform evolutionary rates that have been reported for multiple *S. aureus* clonal lineages (Table 1). Interestingly, a higher evolutionary rate had been found in one study on clonal lineage ST239, with confidence intervals not overlapping with those from most other lineages (Table 1). Our results suggest this may be caused by an elevated, initial mutation rate in some strains affiliated to this population (Fig. 1). The observed ratio of annual base-substitution rates to spontaneous mutation rates measured in vitro requires the bacteria to continuously pass several thousand cell divisions per year on average, corresponding to generation times of < 2 h, which is consistent with a recent estimate [32].

Our results suggest rapid and continuous bacterial cell replication particularly during nasal colonization, rather than infection. This is because annual evolutionary rates and inferred replication rate estimates represent average values for large populations, each of which had been sampled for population genomic analyses over several years, from numerous individual hosts, and across large geographic regions [34, 35]. Base substitution rates had been inferred from branch lengths in phylogenetic trees, which represented time spans of several years. Over long time periods, nasal colonization is the dominant lifestyle of *S. aureus*, as it commonly persists in individuals for many years, whereas infections are rare and most commonly short-lived. Therefore, evolutionary rates mostly represent asymptomatic colonization, even though the majority of genome-sequenced isolates may have been collected from infected patients.

The ratio of sequencing coverage between the origin and terminus of replication (peak-to-trough ratio, PTR) was recently proposed as a proxy for measuring bacterial growth rates in vivo [8, 9]. In our study, metagenomic sequencing yielded PTR > 1.2 for all ten nasal swab samples that had tested positive for *S. aureus* colonization, confirming that *S. aureus* was actively replicating [8]. The median PTR was 1.54, similar to previously reported PTR from bacteria in oral samples [10]. While PTR was used before to assess bacterial growth and compare it among different samples and across bacterial species [8, 9], a calibration of PTR to yield generation-time estimates from metagenomic sequence data had not previously been attempted. Our comparison of PTR from nasal samples to PTR from a culture of *S. aureus* suggested that generation times in vivo had been between two and four hours for 80% of the nasal swabs, and six to seven hours for two of the samples (median generation time, 3.4 h). Hence, the majority of calibrated PTR measurements suggested about two-fold slower cell division rates than inferred from the ratio of evolutionary rate to mutational rate.

The point measurements of growth from a small number of nasal swabs might deviate from the long-term, large-population, average growth rate inferred from the species’ evolutionary rate because neither method captured spatial and temporal fluctuations of the bacterial growth rate, which certainly occur within colonized individuals [49, 50]. After all, even the PTR approach yielded average measurements from population samples of bacteria, whereas replication rates may have been heterogeneous at a single cell level [51, 52]. Standard nasal swabbing, which was applied here, reached only about 2 cm into the nostrils, and it is conceivable that conditions for bacterial cell division are more favorable deeper in the nasal cavity, due to increased moisture and temperature [50]. In addition, shedding dynamics on the posterior nasal mucosa are higher than on the squamous epithelia of the anterior nares [14]. For sampling bacteria from the posterior nasopharynx, however, nasal washing or brushing would be necessary, which is considerably more uncomfortable for participants than nasal swabbing. Recruitment of volunteers would therefore be more difficult and anonymous self-sampling would not be readily possible.

The spontaneous mutation rate of an organism is commonly considered a near-constant trait [45, 53]. Therefore we assumed that the spontaneous mutation rate measured in vitro was a reasonably accurate approximation of the mutation rate in vivo. Some dependency of the mutation rate on environmental conditions cannot be fully excluded, however, and if the mutation rate was higher in the nose than in vitro, our inference would overestimate the in-vivo cell division rate. For example, exposure to antibiotics at subinhibitory levels was reported to increase mutagenesis either through bacterial production of reactive oxygen species that may directly damage DNA [54], or through a cellular stress response that results in upregulation of a DNA polymerase with reduced replication fidelity [55]. However, even though the exposure of bacteria to low concentrations of antibiotics is a plausible scenario due to widespread usage of antibiotic drugs, it is not known if such pollution is sufficiently pervasive to have a measurable effect on average mutation rates in natural *S. aureus* populations [56]. Such ‘stress-induced mutagenesis’ was even proposed as a general evolutionary strategy [57], but this concept is controversial on both, experimental and theoretical grounds [45, 58–60]. Commonly, cultivation conditions were reported to have little effect on the bacterial mutation rate [42, 44, 61]. Indeed, the fairly good match between our generation time estimates based on evolutionary rates and those directly measured in nasal swabs suggests that *S. aureus* mutation rates measured in the laboratory were not very different from mutation rates occurring in vivo.

### Nutrient supply in the nasal cavity

Our replication rate estimates are consistent with previously reported in vitro measurements of *S. aureus* growth in human nasal secretions [62]. However, the maximum staphylococcal growth rate in the human nasal cavity will depend on the local flux of nutrients in relation to the density of bacteria. Human nasal secretions contain amino acids, glucose, and carboxylic acids at a total concentration of 400 mg/l [20]. In a previously reported continuous culture experiment, nutrients from 1 ml of a synthetic growth medium (SNM) mimicking the composition and concentrations of low molecular weight substances in nasal mucus supplied one doubling of 2 × 10^8^ *S. aureus* cells [20]. About 20 to 40 ml of mucus gets secreted in the nose daily and transported to the pharynx [17]. Hence, if the low molecular weight organic substances contained in nasal secretions could be consumed completely, they would suffice for a population of 2 × 10^8^ *S. aureus* cells to perform 20 to 40 cell divisions per day, provided that the population size stayed constant.

The respiratory tract provides nutrients for microbial growth at lower concentrations than the gastrointestinal tract and, accordingly, supports much lower bacterial densities [63]. The precise number of *S. aureus* cells that colonize a human nasal cavity has not been determined. Swabbing of a few square centimeters of the anterior nose usually yields 10^2^ to 10^6^ *S. aureus* colony-forming units [64, 65] or equivalent 16S ribosomal RNA gene counts [66], respectively. The total surface area in the human nose is about 160 cm^2^ [17], and assuming a uniform colonization density across this entire area [67], a colonized nasal cavity in total may carry 10^4^ to 10^8^ *S. aureus* cells. Hence, the low-molecular weight organic substances in nasal mucus will supply ample nutrients for > 20 doublings of the entire nasal *S. aureus* population per every 24 h.

In addition, nasal mucus contains large amounts of macromolecular mucin glycoproteins. Mucins are covered with abundant and complex oligosaccharide structures [68], at least some of which can also serve as a carbon source for *S. aureus* [22]. While other species of bacteria thrive in the nose, too, and may compete for nutrients, *S. aureus* usually predominates the nasal bacterial communities in colonized individuals [66].

### Continuous cell division implies high rate of removal

For the *S. aureus* population size to stay constant over time, the continuous growth implies simultaneous, equally rapid removal. Various mechanisms may contribute to the death of *S. aureus* cells, including phagocytosis by neutrophils [14], killing by human-derived antibacterial peptides [19] or by bacteria-derived antimicrobial compounds [69], or lysis by bacteriophages. Mucociliary clearance, however, is the predominant force for the removal of bacteria and other particles from the nasal cavity [17]. Any particles trapped in the nasal mucus have a half-life to removal of only 20 min [17]. Mucus effectively binds and transports bacteria, including *S. aureus* [70], ensuring that contact of microorganisms with epithelial cells is rare [68]. While adherence to the epithelium is needed to sustain colonization [14, 16], only a small portion of the bacterial population may be adherent, and when adherent cells divide, daughter cells move into the mucus layer.

We found that generation times of *S. aureus* during nasal colonization were between approximately 80 min (long-term average) and 200 min (median from metagenomic sequencing of DNA from anterior nares). Similar generation times (104–161 min) had been determined previously for *Streptococcus pneumoniae* in the nasopharynx of experimentally infected mice [4]. Growth during colonization increases bacterial density and facilitates transmission to another host [63]. Staphylococcal growth will also enhance resistance to colonization by competitors, ensuring that all available receptors remain occupied and nutrients get consumed. Continuous growth may even be required to replenish the adherent population, since the epithelium is also shed, albeit at a lower rate than the mucus [14]. This ‘model’ is similar to one previously proposed for the stomach bacterium *Helicobacter pylori* [1], even though cilia drive the movement of mucus in the respiratory tract, whereas in the gastrointestinal tract, mucus is continually removed by movement of the luminal contents [68]. The balance between bacterial growth and removal likely will affect respiratory health.

### Colonization versus infection

It is well documented that *S. aureus* can reach even shorter doubling times (< 1 h) under favorable conditions in nutrient-rich growth broth (Fig. 3a) or in isolated nasal secretions in vitro [62]. In contrast, little is known currently about growth rates of *S. aureus* during infection. Extremely slow growth (median generation time, 2 days) of *S. aureus* was measured in expectorated sputum from cystic fibrosis patients [51]. Limited oxygen availability in viscous sputum and continuous antibiotic treatment during chronic infection were suspected major growth constraints [51]. Generally, decreased staphylococcal growth rates during chronic infections are thought to hamper successful antibiotic treatment [71]. In contrast, acute infections with *S. aureus* may progress very dynamically. In a mouse sepsis model, staphylococcal invasion was characterized by severe bottlenecks in the bacterial population due to the innate immune response, which were then followed by massive clonal expansions, with up to 100,000-fold increases of the number of *S. aureus* colony-forming units in kidney abscesses within 70 h [72]. Such growth roughly corresponds to a generation time of approximately 4 h (not accounting for bacterial death). Hence, the cell division rates which we determined for nasal colonization in most cases were higher than the fastest documented (net) rates during fulminant disease progression. Even though nutrient concentrations in nasal mucus are considerably lower than in blood plasma or sputum [20], conditions in the nasal environment may still be more favorable for growth due to the rich oxygen supply. Future measurements of in-vivo replication rates of *S. aureus* during diverse courses of infection might improve our understanding of the dynamic ecology of colonization and infection, and associated metabolic transitions.

## Conclusions

We here report absolute generation times for a commensal bacterium during natural colonization of the human host. We provide two lines of genomic evidence which concordantly indicate that *S. aureus* cells in the human nasal cavity replicate every one to three hours, on average. Our results document that *S. aureus* is well adapted to life in the constantly shedding, floating environment of the upper respiratory tract, and that nasal colonization is characterized by a highly dynamic equilibrium between bacterial growth and removal. We provide basic growth rate data that will inform mathematical modelling of the dynamic microbial ecology of the human upper respiratory tract, which eventually may enable the development of novel diagnostics to predict the occasional transition from symptom-free carriage to infectious disease [2]. Finally, our data suggest that the lifestyle of a bacterial pathogen may have a major impact on its rate of molecular evolution.

## Methods

### Bacterial strains and growth conditions

Bacterial isolates used in this work are listed in Additional file 1: Table S1. *S. aureus*, *E. coli* and *K. pneumoniae* were cultivated in tryptic soy yeast-extract broth (TSY, medium 92, www.dsmz.de) at 37 °C.

### Fluctuation analyses

Bacterial overnight cultures were inoculated into TSY broth at 10^5^ CFU/ml. For each experiment, at least 30 independent 200 μl volume cultures were incubated in an Infinite 200 PRO plate reader (TECAN) with continuous shaking at 37 °C. Incubation time was varied between 5 h and 8 h to reach final cell concentrations of 10^8^ CFU/ml for each of the bacterial species, determined by serial dilution in phosphate-buffered saline (PBS) and plating on drug-free TSY agar of 4–6 cultures from each experiment. In addition, 100 μl from each culture was plated on TSY agar containing rifampicin (Sigma) at 100 μg/ml and incubated at 37 °C for 48 h. Each fluctuation assay was performed in duplicate.

The expected number of rifampicin resistance-causing mutations per culture (*m*) was calculated using the maximum-likelihood estimator applying newton.LD.plating function from the rSalvador package v1.7 for *R* [73], which accounts for plating efficiency (i. e., the portion of culture plated, which was 0.5 in our case). Rates of rifampicin resistance were calculated by dividing *m* by the average final cell number (CFU, see above). Statistical comparisons were carried out by using the likelihood ratio test (LRT.LD.plating function from rSalvador), which accounts for differences in final cell numbers, and *P* values were corrected by the Benjamini-Hochberg method [74].

The number and types of distinct mutations causing rifampicin resistance was determined by PCR amplification and Sanger-sequencing of *rpoB* gene fragments from 100 independent, rifampicin-resistant *S. aureus* mutants from our fluctuation experiments. Oligonucleotide primers are listed in Additional file 6: Table S4. Assembly of sequencing raw data and comparative sequence analysis was carried out by using the software Sequencher (available at http://www.genecodes.com/). A mutation rate μ_FA_ per nucleotide was then calculated by dividing the rate of phenotypic rifampicin resistance by the number of distinct mutations causing rifampicin resistance. Experiments and analyses were performed in the same way for *S. aureus*, *E. coli* and *K. pneumoniae*.

### Mutation accumulation experiments

Mutation accumulation experiments were carried out as described previously [41]. Briefly, for each of two *S. aureus* strains (04–02981 and HO 5096 0412), 70 mutation accumulation lines were obtained from single colonies on agar plates and streaked on TSY agar and incubated at 37 °C for 24 h. Every 24 h, a single colony was picked from each line and streaked on a new agar plate. Lines from strain 04–02981 were passaged 58 times and lines from strain HO 5096 0412 were passaged 77 times, and then colonies from each line were subjected to Illumina genome sequencing. To estimate the number of generations that had passed to form bacterial colonies, we enumerated the number of *S. aureus* cells in single colonies by suspending entire colonies in phosphate-buffered saline and plating dilutions on TSY agar. The mutation rate μ_MA_ was calculated as $\mu \mathrm{MA}=\frac{m}{\sum_{i=1}^n{\mathrm{N}}_{\mathrm{i}}\ x\ {\mathrm{T}}_{\mathrm{i}}}$, where *m* is the total number of mutations from all lines of a strain, *n* is the number of lines, *N*_*i*_ is the number of nucleotide sites, and *T* is the number of cell divisions that a line had passed [42]. Confidence intervals were calculated from a Poisson distribution by using the *R* function poisson.test (R version 3.4.0).

### In vitro growth experiment with *Staphylococcus aureus*

An overnight-culture of *S. aureus* 15–00140 (nasal isolate) was inoculated into tryptic soy yeast-extract broth at 9.2 × 10^6^ CFU/ml, and 200 μl cultures were incubated in an Infinite 200 PRO plate reader (TECAN) with continuous shaking at 37 °C. The optical density at 600 nm was monitored over time and six replicate 200 μl samples were harvested every 30 min for 7.5 h. Temporal in vitro generation times *g*_*i*_ at sampling time points *t*_*i*_ were calculated as $$ {g}_i=\frac{t_{i+30\ \mathit{\min}}-{t}_{i-30\ \mathit{\min}}}{\mathit{\log}2\left(\raisebox{1ex}{${OD}_{t_{i+30\ \mathit{\min}}}$}\!\left/ \!\raisebox{-1ex}{${OD}_{t_{i-30\ \mathit{\min}}}$}\right.\right)} $$.

### Nasal sampling

Nasal swab samples were collected anonymously from 48 adult volunteers by self-swabbing their anterior nares using dry, sterile Purflock swabs (MWE Medical Wire, UK). Sampling swabs were promptly placed in a − 20 °C freezer until further processing. Voluntary participants were recruited among employees of the Leibniz Institute DSMZ, Braunschweig. Participants confirmed they were not taking any antibiotic medication.

### DNA extraction, quantitative PCR, and genome sequencing

Material from nasal swabs was suspended in 1 ml phosphate-buffered saline (pH 7.4; Roth) and DNA was extracted by using the QIAamp DNA Microbiome kit (Qiagen) according to the manufacturer’s instructions. Genomic DNA from *S. aureus* cultures was extracted using the DNeasy Blood & Tissue kit (Qiagen) with 1–2 crystals of lysostaphin added to the lysis buffer per each sample.

Quantitative PCR (qPCR) was applied to estimate the yield of extracted DNA from both, *S. aureus* and the human host. PCR primers targeted the staphylococcal gyrase B gene [75] and the human interferon beta-1 gene (Additional file 6: Table S4). qPCR was performed in duplicate, in 20 μl reactions each, containing 2 μl of DNA, 1 μl of each specific primer (10 pmol), and 10 μl of 2 × LightCycler 480 SYBR Green I Master (Roche). Thermal cycling on a LightCycler 480 Real Time PCR system (Roche) consisted of 5 min. at 95 °C followed by 45 cycles of 10 s. at 95 °C, 20 s. at 60 °C and 20 s. at 72 °C. DNA from *S. aureus* SH1000 (a gift from Simon J. Foster, University of Sheffield, UK) and HELA cell line ACC 57 (www.dsmz.de), quantified with the Qubit dsDNA BR Assay Kit (Thermo Fisher Scientific), was used for calibration. Illumina sequencing libraries were prepared by using a cost-effective protocol based on Nextera XT chemistry as described previously [76] and sequenced on an Illumina NextSeq 500 machine using a Mid-Output kit (Illumina) with 300 cycles.

### Bioinformatic analyses and prediction of doubling times from sequencing coverage

Sequencing reads from mutation accumulation lines (sequencing coverage, ≥40-fold) were mapped to the reference genome sequences from *S. aureus* strains 04–02981 (CP001844) or HO 5096 0412 (NC_017763), respectively, by using BWA-MEM (v0.7.12) at default settings and mutations were identified by using VarScan2 (v2.3) as described previously [76]. Briefly, Samtools (v0.1.19) was used to process BAM files, retaining sequencing reads with a minimum mapping quality (−Q) of 30, and VarScan2 parameters for variant calling were set to mincoverage = 10, minfreqforhom = 0.75, minvarfrequency = 0.8, minreads2 = 6, *p*-value = 0.01, minavqual = 20, strandfilter = 1.

Gene-content differences among genome sequences from strains NCTC11939 (available at ftp://ftp.sanger.ac.uk/pub/project/pathogens/NCTC3000/datalinks_manual/ERS798843.gff), TW20 (FN433596–8) and HO 5096 0412 (NC_017763) were determined based on a pan genome analysis (without a core alignment) by using Roary 3.11.2 (software available at https://sanger-pathogens.github.io/Roary/).

DNA sequencing reads from nasal swab samples and from the growth experiment with *S. aureus* strain 15–00140 were mapped to the reference genome sequence from *S. aureus* MSSA476 (acc. no. NC_002953) by using BWA-MEM version 0.7.12 at default settings [77]. To test for the effect of the reference sequence applied, sequencing reads were also mapped to genome sequences from *S. aureus* strains COL (NC_002951), N315 (NC_002745), HO 5096 0412 (NC_017763), S0385 (NC_017333). Resulting BAM files were converted to SAM format by using SAMtools version 0.1.19 [78], retaining reads mapped in pairs with a mapping quality ≥q30. Peak-to-through ratios (PTR) of the sequencing coverage along the *S. aureus* genome were determined by using the bPTR Python script v1.10 [9].

A polynomial regression of 2nd order was used to predict doubling times from nasal swab PTR values. The best model fit of PTR values to doubling times from the *S. aureus* laboratory culture was determined by model comparison using *R* (stats package v3.5.0). A polynomial fit of 2nd order yielded the lowest corrected Akaike information criterion value (AICc, 20.5) [79] in comparison to a polynomial fit of 3rd order (AICc, 22.0) or to fits using exponential (AICc, 23.7) or gamma distributions (AICc, 26.1). Morever, the polynomial fit of 2nd order yielded the largest adjusted R^2^ value (0.89). The observed residuals from the polynomial fit of 2nd order did not deviate significantly from expected residuals under normality (Kolmogorov-Smirnov test, *p* = 0.71).

## Additional files

Additional file 1: **Table S1.** Bacterial isolates. Metadata, sources, and references to literature. (PDF 529 kb)

Additional file 2: **Table S2.** Mutations conferring rifampicin resistance. Nucleotide positions in *rpoB* gene, nucleotide changes, and amino acid changes. (PDF 497 kb)

Additional file 3: **Table S5.** Mutations in strain 04–02981. Mutations detected in mutation accumulation experiment. (PDF 431 kb)

Additional file 4: **Table S6.** Mutations in strain HO 5096 0412. Mutations detected in mutation accumulation experiment. (PDF 428 kb)

Additional file 5: **Table S3.** qPCR results. Quantitative PCR results for 46 nasal swab samples. (PDF 513 kb)

Additional file 6: **Table S4.** PCR primers. Oligonucleotide sequences and product lengths. (PDF 547 kb)

## Abbreviations

AICc: Akaike information criterion value
CFU: Colony-forming units
C_t_ value: Treshold cycle
PBS: Phosphate-buffered saline
PTR: Peak-to-trough ratio
qPCR: Quantitative polymerase chain reaction
TSY: Trytic soy yeast-extract
