# Supplementary material for: Rapid cell division of Staphylococcus aureus during colonization of the human nose
Source: BMC Genomics. 2019 Mar 20;20:229. doi: 10.1186/s12864-019-5604-6 (PMC6425579; doi:10.1186/s12864-019-5604-6)
Supplement: Supplementary file 1 — Table S1. Bacterial isolates. Metadata, sources, and references to literature. (PDF 529 kb) [file 12864_2019_5604_MOESM1_ESM.pdf]

Suppl. Table S1. Bacterial isolates.

Staphylococcus aureus.

Isolate	Sequence type	Clonal complex	Methicillin resistance	Country of origin	Year of isolation	Synonymous name	Strain collection	Literature
11-02810	ST22	22	yes	UK	2005	HO 5096 0412	Robert Koch Institute	Holden et al. 2013, Genome Research 23: 653
07-02389	ST22	22	no	Germany	2007		Robert Koch Institute	Kurt et al. 2009, J. Clin. Microbiol. 47: 577
98-01907	ST36	30	yes	Germany	1998		Robert Koch Institute	Kurt et al. 2009, J. Clin. Microbiol. 47: 577
06-01300	ST30	30	no	Germany	2006		Robert Koch Institute	Kurt et al. 2009, J. Clin. Microbiol. 47: 577
04-02981	ST225	5	yes	Germany	2004		Robert Koch Institute	Nübel et al. 2010, PLOS Pathogens 6: e1000855
06-00966	ST5	5	no	Germany	2006		Robert Koch Institute	Kurt et al. 2009, J. Clin. Microbiol. 47: 577
04-03094	ST398	398	yes	Germany	2004		Robert Koch Institute	Kurt et al. 2009, J. Clin. Microbiol. 47: 577
06-03005	ST398	398	no	Germany	2006		Robert Koch Institute	Kurt et al. 2009, J. Clin. Microbiol. 47: 577
08-01911	ST1	1	yes	USA	1998	MW2	Robert Koch Institute	Baba et al. 2002, Lancet 359: 1819
05-02040	ST8	8	yes	Germany	2005		Robert Koch Institute	Kurt et al. 2009, J. Clin. Microbiol. 47: 577
04-00241	ST8	8	no	Germany	2004		Robert Koch Institute	Kurt et al. 2009, J. Clin. Microbiol. 47: 577
93-00635	ST239	8	yes	Germany	unknown		Robert Koch Institute	Kurt et al. 2009, J. Clin. Microbiol. 47: 577
NCTC11939	ST239	8	yes	UK	1986		NCTC	Marples et al. 1985, J. Hosp. Infect. 6: 342

Klebsiella pneumoniae.

Isolate	Sequence type	Country of origin	Year of isolation	Synonymous name	Strain collection	Literature
E909-1	ST258	Germany	2013		University Clinic Cologne	
U6064-2	n. d.	Germany	2013		University Clinic Cologne	
SG0677	ST258	Germany	2007		University Clinic Frankfurt	
SG1262	ST258	Germany	2011		University Clinic Frankfurt	
SG1304	ST258	Germany	2011		University Clinic Frankfurt	
SG1377	ST258	Germany	2011		University Clinic Frankfurt	
SG1984	ST258	Germany	2014		University Clinic Frankfurt	
234/12	ST512	Germany	2011	234/12	Robert Koch Institute	Becker et al. 2015, Genome Ann. 3: e00742-15

Escherichia coli.

Isolate	Sequence type	Country of origin	Year of isolation	Synonymous name	Strain collection	Literature
TO143	ST131	Germany			University Clinic Cologne	
TO6	ST10	Germany			University Clinic Cologne	
TO196	ST131	Germany			University Clinic Cologne	
TO216	ST131	Germany			University Clinic Cologne	
