# Supplementary material for: Rapid cell division of Staphylococcus aureus during colonization of the human nose
Source: BMC Genomics. 2019 Mar 20;20:229. doi: 10.1186/s12864-019-5604-6 (PMC6425579; doi:10.1186/s12864-019-5604-6)
Supplement: Supplementary file 2 — Table S2. Mutations conferring rifampicin resistance. Nucleotide positions in rpoB gene, nucleotide changes, and amino acid changes. (PDF 497 kb) [file 12864_2019_5604_MOESM2_ESM.pdf]

Suppl. Table S2. Mutations conferring rifampicin resistance.

Nucleotide position*	Mutation	Amino acid change
1402	C>G	Q468E
1402	C>A	Q468K
1403	A>G	Q468R
1403	A>T	Q468L
1411	G>A	D471N
1413	C>A	D471E
1430	C>T	A477V
1430	C>A	A477D
1441	C>T	H481Y
1441	C>A	H481N
1441	C>T	H481Y
1442	A>C	H481P
1450	C>A	R484S
1450	C>T	R484C
1451	G>A	R484H
1457	C>T	S486L
1456, 1457	C>A	S486K

* nucleotide position in *rpoB* gene from *S. aureus* NCTC 8325
