# Supplementary material for: Rapid cell division of Staphylococcus aureus during colonization of the human nose
Source: BMC Genomics. 2019 Mar 20;20:229. doi: 10.1186/s12864-019-5604-6 (PMC6425579; doi:10.1186/s12864-019-5604-6)
Supplement: Supplementary file 3 — Table S5. Mutations in strain 04–02981. Mutations detected in mutation accumulation experiment. (PDF 431 kb) [file 12864_2019_5604_MOESM3_ESM.pdf]

Suppl. Table S5. Mutations in strain 04-02981 (mutation accumulation experiment).

Genomic position	DNA change	amino acid change	open reading frame
13475	C->T	T->I	SA2981_0009
74534	C->T	I->I	SA2981_0071
108125	G->T	K->N	SA2981_0101
111740	C->T	T->I	SA2981_0103
114448	C->T	NC	
114758	G->A	R->R	SA2981_0106
148026	G->T	NC	
218464	C->T	R->W	SA2981_0192
256396	A->G	I->I	SA2981_0224
261979	C->T	R->*	SA2981_0228
342860	A->G	I->V	SA2981_0298
370044	A->G	L->L	SA2981_0322
406846	C->T	NC	
425007	G->T	NC	
429094	A->G	V->V	SA2981_0386
431423	A->G	T->A	SA2981_0388
501567	G->A	V->I	SA2981_0453
549791	C->T	NC	
557942	G->A	G->G	SA2981_0496
610509	T->C	NC	
666913	T->G	NC	
666918	T->A	NC	
666924	A->T	NC	
666925	A->T	NC	
666926	T->G	NC	
719227	G->A	A->T	SA2981_0645
780239	T->A	NC	
848329	T->C	D->D	SA2981_0766
848359	C->A	D->E	SA2981_0766
848364	G->A	G->D	SA2981_0766
880055	T->G	P->P	SA2981_0807
888279	C->T	T->I	SA2981_0828
898737	A->G	M->V	SA2981_0849
925602	C->T	A->V	SA2981_0878
977482	G->A	P->P	SA2981_0925
1000571	C->T	C->C	SA2981_0947
1001563	A->G	G->G	SA2981_0948
1001569	G->T	S->S	SA2981_0948
1007430	G->T	E->D	SA2981_0954
1073818	C->T	S->L	SA2981_1016
1080341	C->T	A->V	SA2981_1025
1083920	C->A	A->E	SA2981_1027
1124983	T->C	F->L	SA2981_1070
1181463	T->C	N->N	SA2981_1126
1225111	C->T	S->S	SA2981_1166
1235536	T->C	A->A	SA2981_1176
1238561	C->T	A->V	SA2981_1178
1243544	C->T	Q->*	SA2981_1184
1270512	C->T	P->L	SA2981_1206
1344584	G->A	NC	
1367801	G->A	S->N	SA2981_1289
1390533	C->G	P->A	SA2981_1308
1414250	G->A	V->V	SA2981_1328
1435556	A->G	NC	
1459239	A->G	NC	
1459438	G->T	E->E	SA2981_1370
1473884	G->T	A->A	SA2981_1389
1501943	G->T	T->T	SA2981_1390
1520783	T->G	V->G	SA2981_1405
1526546	G->A	A->A	SA2981_1410
1551250	A->T	N->I	SA2981_1435
1565265	G->A	NC	
1695229	G->A	NC	
1709673	C->A	H->H	SA2981_1597
1823575	T->C	R->R	SA2981_1698
1837803	C->T	K->K	SA2981_1710
1893331	G->A	I->I	SA2981_1763
1900590	A->T	NC	
1915475	C->T	T->T	SA2981_tRNA25
1973970	G->A	D->N	SA2981_1833
2070705	G->A	K->K	SA2981_1945
2084292	C->T	G->G	SA2981_1966
2121383	C->T	A->V	SA2981_1998
2195039	C->T	A->A	SA2981_2064
2216035	G->A	S->S	SA2981_2084
2217444	G->A	G->D	SA2981_2086
2258838	G->A	D->D	SA2981_2112
2267217	G->C	Q->H	SA2981_2119
2273790	T->C	Y->Y	SA2981_2125
2281182	C->T	T->T	SA2981_2131
2286585	T->C	NC	
2300595	G->A	S->S	SA2981_2150
2308935	G->A	N->N	SA2981_2162
2319252	C->A	P->P	SA2981_2185
2321941	T->C	NC	
2413869	T->C	Y->Y	SA2981_2284
2531856	G->A	A->A	SA2981_2389
2573979	C->T	NC	
2585567	G->A	N->N	SA2981_2439
2608567	C->A	NC	
2652746	G->A	Q->Q	SA2981_2498
2655591	G->A	L->L	SA2981_2501
2691365	C->T	S->L	SA2981_2541
2704012	C->A	I->I	SA2981_2551
2716642	T->C	NC	
2725742	C->T	V->V	SA2981_2569
2800865	C->T	H->Y	SA2981_2630

NC, non-coding
genomic positions and open reading frames in reference genome from strain 04-02981 (CP001844).
