# Supplementary material for: Rapid cell division of Staphylococcus aureus during colonization of the human nose
Source: BMC Genomics. 2019 Mar 20;20:229. doi: 10.1186/s12864-019-5604-6 (PMC6425579; doi:10.1186/s12864-019-5604-6)
Supplement: Supplementary file 4 — Table S6. Mutations in strain HO 5096 0412. Mutations detected in mutation accumulation experiment. (PDF 428 kb) [file 12864_2019_5604_MOESM4_ESM.pdf]

Suppl. Table S6. Mutations in strain HO 5096 0412 (mutation accumulation experiment).

Genomic position	DNA change	amino acid change	open reading frame
8521	A->G	Q->R	SAEMRSA15_00060
20545	G->A	G->D	SAEMRSA15_00150
38158	T->A	NC	
47368	C->T	G->G	SAEMRSA15_00360
61353	G->A	E->E	SAEMRSA15_00470
85597	C->T	D->D	SAEMRSA15_00690
103293	C->A	NC	
115784	G->A	NC	
145809	G->A	D->N	SAEMRSA15_01180
160543	A->G	D->G	SAEMRSA15_01330
175002	G->A	V->I	SAEMRSA15_01440
185198	G->T	NC	
193779	C->T	R->C	SAEMRSA15_01560
209842	T->C	NC	
212317	A->C	G->G	SAEMRSA15_01690
215035	G->T	G->V	SAEMRSA15_01720
219613	G->A	A->T	SAEMRSA15_01760
257257	T->G	F->C	SAEMRSA15_02030
353755	G->T	NC	
381949	A->T	I->F	SAEMRSA15_03150
391990	C->A	NC	
517781	A->G	NC	
533059	C->A	NC	
549305	G->A	G->R	SAEMRSA15_04630
581345	G->T	D->Y	SAEMRSA15_04880
585823	C->T	D->D	SAEMRSA15_04890
585829	T->C	D->D	SAEMRSA15_04890
585835	T->C	D->D	SAEMRSA15_04890
585883	C->T	D->D	SAEMRSA15_04890
586837	A->G	NC	
587374	C->G	T->R	SAEMRSA15_04900
618643	A->G	N->N	SAEMRSA15_05220
653943	C->T	Q->Q	SAEMRSA15_05570
670953	C->T	T->I	SAEMRSA15_05740
705607	C->T	A->V	SAEMRSA15_06100
752452	A->G	S->S	SAEMRSA15_06530
781685	T->G	F->V	SAEMRSA15_06800
800919	T->C	L->S	SAEMRSA15_06950
862547	C->T	T->M	SAEMRSA15_07590
1014602	G->A	G->R	SAEMRSA15_09000
1021765	T->C	L->S	SAEMRSA15_09050
1030495	G->T	V->F	SAEMRSA15_09140
1079574	T->A	N->K	SAEMRSA15_09620
1084773	G->A	G->D	SAEMRSA15_09690
1093133	G->A	E->E	SAEMRSA15_09740
1182522	C->T	G->G	SAEMRSA15_10620
1227295	T->G	NC	
1276043	C->A	A->E	SAEMRSA15_11410
1279212	C->T	NC	
1387254	T->A	H->H	SAEMRSA15_12530
1463089	G->C	I->I	SAEMRSA15_12980
1541985	G->A	A->A	SAEMRSA15_13710
1545101	C->T	L->L	SAEMRSA15_13740
1548830	T->A	V->V	SAEMRSA15_13790
1591989	G->A	N->N	SAEMRSA15_14330
1748956	C->A	R->R	SAEMRSA15_15940
1801078	G->A	K->K	SAEMRSA15_16350
1813324	G->A	R->Q	SAEMRSA15_16420
1823134	G->T	K->K	SAEMRSA15_16490
1861604	C->A	NC	
1867268	C->A	V->V	SAEMRSA15_16840
1875249	C->T	P->L	SAEMRSA15_16950
1895713	G->A	NC	
1950877	C->G	NC	
2000973	G->A	Q->Q	SAEMRSA15_18120
2020764	G->A	G->G	SAEMRSA15_18310
2207926	C->A	K->K	SAEMRSA15_20360
2219465	C->T	NC	
2247190	C->T	NC	
2257605	C->A	NC	
2341114	G->T	NC	
2358122	G->A	K->K	SAEMRSA15_21820
2361410	A->G	NC	
2371814	G->A	G->D	SAEMRSA15_21960
2408137	G->T	NC	
2479306	C->A	K->K	SAEMRSA15_22970
2495388	A->G	NC	
2505456	T->C	D->D	SAEMRSA15_23230
2510957	C->T	V->V	SAEMRSA15_23290
2537111	T->C	NC	
2549486	G->A	R->H	SAEMRSA15_23590
2590571	G->A	A->A	SAEMRSA15_23980
2598445	C->T	NC	
2602454	G->A	A->A	SAEMRSA15_24070
2605390	G->A	NC	
2607131	T->C	V->V	SAEMRSA15_24100
2629627	G->A	E->E	SAEMRSA15_24340
2700009	A->G	T->T	SAEMRSA15_25040
2722132	G->A	NC	
2813206	C->A	NC	
2831424	G->T	NC	

NC, non-coding
genomic positions and open reading frames in reference genome from strain HO 5096 0412 (NC_017763).
