# Supplementary material for: Rapid cell division of Staphylococcus aureus during colonization of the human nose
Source: BMC Genomics. 2019 Mar 20;20:229. doi: 10.1186/s12864-019-5604-6 (PMC6425579; doi:10.1186/s12864-019-5604-6)
Supplement: Supplementary file 5 — Table S3. qPCR results. Quantitative PCR results for 46 nasal swab samples. (PDF 513 kb) [file 12864_2019_5604_MOESM5_ESM.pdf]

Suppl. Table S3. qPCR results.

nasal sample	DNA concentration (ng/µl)	***C t* S. aureus (*gyrB*)**	***C t* human (*IFNB1*)**	sequenced
1	0.16	26	32	yes
2	0.12	27	34	yes
3	0.04	39	35	no
4	0.08	>40	33	no
5	0.05	>40	34	no
6	0.09	33	33	yes
7	0.07	40	33	no
8	9.93	39	24	no
9	n. d.	>40	>40	no
10	n. d.	32	38	yes
11	0.31	>40	31	no
12	0.05	>40	35	no
13	0.04	29	35	yes
14	0.06	28	34	yes
15	0.11	40	34	no
16	0.06	38	36	no
17	0.04	39	39	no
18	0.25	28	31	yes
19	0.55	31	36	yes
20	0.61	33	34	yes
21	0.03	>40	36	no
22	0.08	>40	33	no
23	0.22	39	31	no
24	n. d.	39	37	no
25	0.05	>40	40	no
26	0.04	39	35	no
27	0.11	34	32	no
28	1.33	40	28	no
29	0.03	40	35	no
30	0.45	40	30	no
31	0.10	40	33	no
32	0.37	27	31	yes
33	0.60	>40	29	no
34	0.28	29	31	yes
35	0.05	>40	34	no
36	0.04	>40	38	no
37	0.06	>40	34	no
38	0.07	>40	34	no
39	0.07	40	34	no
40	0.08	>40	33	no
41	0.10	>40	33	no
42	0.14	40	35	no
43	0.08	>40	34	no
44	0.13	23	33	yes
45	0.29	23	31	yes
46	0.78	22	29	yes

n. d., below detection limit
