# Supplementary material for: Rapid cell division of Staphylococcus aureus during colonization of the human nose
Source: BMC Genomics. 2019 Mar 20;20:229. doi: 10.1186/s12864-019-5604-6 (PMC6425579; doi:10.1186/s12864-019-5604-6)
Supplement: Supplementary file 6 — Table S4. PCR primers. Oligonucleotide sequences and product lengths. (PDF 547 kb) [file 12864_2019_5604_MOESM6_ESM.pdf]

Suppl. Table S4. PCR primers.

Primer name	Sequence	Target gene	Product length (basepairs)	Reference
rpoB_sa_f	AGT CTA TCA CAC CTC AAC AA	*rpoB* from *S. aureus*	702	Aubry-Damon et al. 1998, AAC 42: 2590
rpoB_sa_r	TAA TAG CCG CAC CAG AAT CA			
gyrB_sa_f	ACG CAG GCG ATT TTA CCA T	*gyrB* from *S. aureus*	187	Szafrańska et al. 2014, MBio 5: e01775-14
gyrB_sa_r	CAT CCA CAT CGG CAT CAG T			
infb1_f	GCT CTC CTG TTG TGC TTC TCC	*INFB1* from *H. sapiens*	198	this study
infb1_r	GTC CTC CTT CTG GAA CTG CTG			
